# Persistence of peripheral CD8 + CD28− T cells indicates a favourable outcome and tumour immunity in first-line HER2-positive metastatic breast cancer

**DOI:** 10.1038/s41416-024-02610-0

**Published:** 2024-03-22

**Authors:** Xiaoran Liu, Xiangming Cheng, Feng Xie, Kun Li, Yongcan Shi, Bin Shao, Xu Liang, Fengling Wan, Shidong Jia, Yue Zhang, Yiqiang Liu, Huiping Li

**Affiliations:** 1 Key Laboratory of Carcinogenesis and Translational Research (Ministry of Education/Beijing), Department of Breast Oncology, Peking University Cancer Hospital & Institute, Fu-Cheng road No. 52, Hai-Dian District, Beijing, China; 2 Jin Xiang People’s Hospital, Department of Hematologic Oncology, Jining, Shandong China; 3 Huidu (Shanghai) Medical Sciences, Ltd., Shanghai, China

**Keywords:** Breast cancer, Tumour immunology

## Abstract

**Background:**

The contradictory role of CD8 + CD28− T cells in tumour immunity has been reported, while their biological and clinical significance in HER2-positive metastatic breast cancer (MBC) is still unknown.

**Methods:**

HER2-positive MBC patients with no prior therapy in the metastatic setting were retrospectively recruited at two medical centres. Peripheral CD8 + CD28− T cells (pT_CD8+CD28-_) were detected at baseline and following therapeutic intervals. Progression-free survival (PFS) was compared according to pT_CD8+CD28−_ levels. The molecular features of pT_CD8+CD28−_ and its correlation with tumour immunity were also investigated.

**Results:**

A total of 252 patients were enrolled, and the median follow-up time was 29.6 months. pT_CD8+CD28−_ high at baseline has prolonged PFS compared to pT_CD8+CD28−_ low (*P* = 0.001). Patients who maintained pT_CD8+CD28−_ high had a longer PFS than those who kept pT_CD8+CD28−_ low (*P* < 0.001). The enhanced pT_CD8+CD28−_ level also indicates a longer PFS compared to pT_CD8+CD28−_ low (*P* = 0.025). Here, pT_CD8+CD28-_ was demonstrated as an antigen-experienced effector T cell. Higher IL-2 level (*P* = 0.034) and lower TGF-β level (*P* = 0.016) in the serum and highly infiltrated CD8 + CD28− T cells (*P* = 0.037) were also connected to pT_CD8+CD28−_ high.

**Conclusions:**

High pT_CD8+CD28−_ level is associated with a favourable tumour immunity and a better PFS of HER2-targeting therapy in MBC patients.

## Introduction

The human immune system plays an important role in the progression of cancer [1]. The intimate interaction between lymphocytes and breast cancer development has been reported [2, 3]. Among various subpopulations of lymphocytes, activated CD8+ cytotoxic T lymphocytes (CTLs) serve as a potent mechanism through which the immune system eliminates tumours [4]. The full activation of CTLs requires two fundamental signals: T-cell receptor (TCR) signalling and co-stimulatory signalling [5]. During this process, CD28 is an indispensable molecule needed for initiating co-stimulatory signalling [6]. CD28-mediated co-stimulatory signalling contributes to T-cell survival, proliferation, cytokine production, and metabolism [5]. Alongside the maturation of CTLs, CD28 expression is progressively and irreversibly down-regulated [6]. The CD8 + CD28− T cell (T_CD8+CD28-_) was first identified as a phenotype associated with memory/effector cells [7]. T_CD8+CD28-_ also expresses some natural killer cell-related receptors, which mediate TCR-independent cytotoxicity, suggesting its innate immune property [8]. The increased level of T_CD8+CD28-_ has been shown to be related to normal aging, chronic viral infections, and malignancies [8, 9]. Notably, studies have reported conflicting roles of T_CD8+CD28-_ in various cancers. The high cytotoxic potential of T_CD8+CD28-_ has been found in melanoma, head and neck cancer, cervical cancer and hepatocellular carcinoma [10–13]. On the contrary, the regulatory or suppressive role of T_CD8+CD28−_ has been identified in lung cancer, pleural mesothelioma, and glioblastoma [14–16]. There is now a growing consensus that T_CD8+CD28−_ displays an immunosuppressive function in many other cancers [17]. Currently, only a few studies have reported on the role of T_CD8+CD28−_ in breast cancer. Peripheral T_CD8+CD28-_ (pT_CD8+CD28-_) has been identified as a prognostic factor for progression-free survival (PFS) in metastatic breast cancer (MBC) patients who received chemotherapy or adoptive T-cell therapy [18, 19]. These two studies both suggested a negative prognostic role of pT_CD8+CD28−_. However, the aforementioned studies primarily concentrated on the initial level of pTCD8 + CD28− and regrettably overlooked the dynamic alterations that occur in response to clinical intervention. Besides, the regimens, clinical subtypes, and therapeutic lines of these study cohorts were not strictly confined. These limitations may undermine our understanding of the multifaceted nature of pT_CD8+CD28−_, as our research revealed a varied prognostic significance of peripheral CTL according to molecular subtypes of MBC [20]. Given the aforementioned unresolved issues, we conducted dynamic monitoring of pT_CD8+CD28−_ in HER2-positive (HER2 + ) MBC patients who underwent first-line HER2-targeting therapy. Our objective was to shed light on the biological significance of pT_CD8+CD28−_. Surprisingly, we found a favourable prognostic role for pT_CD8+CD28-_ in HER2-positive MBC, which differs from previous reports.

## Materials and methods

### Patient enrollment and study design

**Fig. 1:**
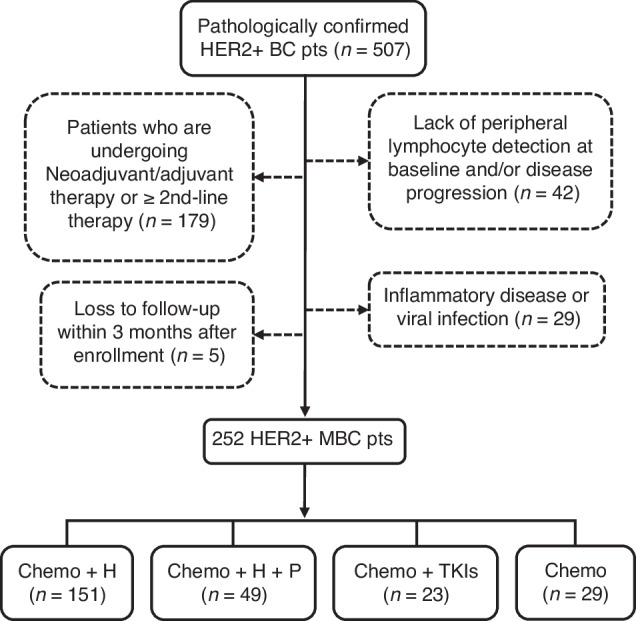
Flow chart of patient selection and classification according to the therapeutic regimens. H trastuzumab, P pertuzumab, TKIs tyrosine kinase inhibitors, pts patients.

BC patients were recruited at Peking University Cancer Hospital and Jin Xiang People’s Hospital between January 2014 and September 2020. The criteria for BC patient selection are summarised in Supplementary Table S1. Oestrogen Receptor (ER) and Progesterone Receptor (PgR) positivity was evaluated by immunohistochemistry (IHC), using antibodies against ER (CONFIRM anti-Oestrogen Receptor (ER)(SP1), Ventana Medical Systems, Inc., Arizona, USA) and PgR (CONFIRM^TM^ anti-Progesterone Receptor (PR)(1E2), Ventana Medical Systems, Inc., Arizona, USA). IHC staining against HER2 (VENTANA anti-HER2/neu (4B5), Roche Diagnostics, Mannheim, Germany) was scored according to intensity as 0, 1+ , 2+ or 3 + ; sample with scores of 0 ~ 1+ was considered HER2 − , while sample with a score of 3+ was considered HER2 + . HER2 amplification of 2+ samples were confirmed by FISH. Eligible HER2 + MBC patients were grouped according to the regimen they received. The peripheral lymphocyte (pL) was detected at baseline and subsequent follow-up visits until failure of first-line treatment. The flow diagram of the cohort selection is shown in Fig. 1. All procedures involving human participants met with the criteria of the Peking University Cancer Hospital ethical committee (Ethic No. 2016KT47) and Jin Xiang People’s Hospital ethical committee (Ethic No. 2021092301).

### Patients’ follow-up

For each enrolled patient, there is one pre-treatment follow-up and then a routine post-treatment follow-up every 2 or 3 therapeutic cycles for imaging-based medical assessments to monitor disease progression. The primary endpoint of this study was PFS, which was defined as the time from the start of therapy to disease progression (PD) or the last date of follow-up. Patients alive without an event as of the PD date were censored at the last study follow-up date (September 30, 2020). Treatment efficacy was evaluated by diagnostic imaging per Response Evaluation Criteria in Solid Tumours (RECIST) 1.1 [21].

### Peripheral lymphocyte (pL) subtype and cytokines detection

Peripheral blood (4 ml) from each patient was drawn into EDTA anticoagulation tubes (Invitrogen, BD). Whole blood (200 μl) was incubated with primary antibodies and subsequently went through haemolysis. Samples were then centrifuged and the supernatant was removed. Finally, the samples were re-suspended twice in PBS and subjected to flow cytometric analysis. Primary antibodies included CD3-PC5/CD4-FITC/CD8-PE (IM1650), CD8-FITC (A07756), CD4-FITC (A07750), CD3-FITC/CD (16 + 56)-PE (A07735), CD19-PC5 (A07771), and CD25-PE (A07774), CD28-PE (IM2071U), CD279 (PD1)-PE (B30634) (all from Beckman Coulter, Inc., CA, USA). Level of pL subtype was expressed as a percentage of the total lymphocytes. Total lymphocytes were selected according to physical characteristics, including volume size and transmissivity. The detection of perforin and granzyme B was conducted according to the manufacturer’s protocol (Qingdao Raisecare Biological Technology Co., Ltd). Directly labelled antibodies against CD3-PerCP (R3901002), CD8-APC (R4601002), CD28 (IM2071U), perforin (R4601002) and granzyme B (R4701002) were purchased from Qingdao Raisecare Biological Technology Co., Ltd. Flow cytometry was performed using Beckman-Coulter FC500 and CXP analysis software (Beckman Coulter, Inc., CA, USA). Each analysis included 10,000 gated events. The gating strategies for each peripheral lymphocyte subtype can be found in Supplementary Fig. S1. The cut-off value of pT_CD8+CD28−_ regarding PFS was determined using X-tile in the training cohort. An additional 79 healthy cases of physical examination between 2014 and 2017 were included as a reference for pL-level analysis.

### Tumour-infiltrating lymphocytes (TILs) evaluation

Two experienced pathologists independently performed the TILs evaluation on full whole-slide sections stained with hematoxylin & eosin. These sections were obtained from needle biopsies of metastatic lesions in patients with HER2 + MBC. TILs were assessed using the guidelines of the international TIL working group [22]. Briefly, stromal TILs were measured as a percentage of immune cells in stromal tissue within the tumour that showed a mononuclear immunological infiltrate. The level of TILs was analysed as a continuous measurement ranging from 0 to 100%.

### Confocal immunofluorescence assay and infiltrated CD8 + CD28− T cell

For immunofluorescent staining, sections were blocked and incubated overnight at 4 °C with primary antibodies. The dilution of the primary antibodies against CD8 (ab237709, Abcam, Cambridge, UK) and CD28 (ab193350, Abcam, Cambridge, UK) was both 1:50. After washing in PBS, sections were incubated with fluorescence-conjugated secondary antibodies at a dilution of 1:200 (ab6717, Abcam, Cambridge, UK; bs-0296P-PE-Cy3, Bioss, Beijing, China) for 1 hour at room temperature. Sections were then washed and counterstained with nuclear dye 4,6-diamino-2-phenylindole (DAPI). The resulting images were visualised and captured on a confocal microscope (FV1200, Olympus, Japan). The infiltrated CD8 + CD28- T cells in tumour tissue were evaluated by two independent pathologists. Every ten fields per slide were selected to calculate the percentage of target cells in all nucleated cells within the tumour nests and tumour stroma. The percentage of infiltrated CD8 + CD28- T cells ranged from 0 to 100%.

### EILSA detection for serum level of IFN-γ, IL-2,TNF-α and TGF-β

The serum samples were collected from patients with empty stomach in the morning. Blood was collected in EDTA tube at 1600 × *g* for 10 min at 4 °C, 1 h after collection. It was then transferred into tubes and kept at −80 °C for further use. Human serum IFN-γ, IL-2, TNF-α and TGF-β antigens were measured using commercially available kits: Human IFN-γ ELISA (EHC102g, QuantiCyto^®^, China), Human IL-2 Instant ELISA^TM^ (BMS221INST, Thermo Scientific Inc., US), Human TNF alpha Instant ELISA^TM^ (BMS2231INST, Thermo Scientific Inc., USA) and Human TGF-β1 ELISA (KGEHC107b, Jiangsu KeyGEN BioTECH Ltd, China) respectively. Assays were performed according to the manufacturer’s protocol. All samples were assayed using an automated immunoassay analyzer AIA-system (TOSOH Corp., Tokyo, Japan).

### Plasma cfDNA extraction and NGS testing

cfDNA was extracted from plasma samples using QIAamp circulating nucleic acid kit. In all, 5–20 ng of the extracted cfDNA were prepared for library construction, including end-repair, dA-tailing, adaptor ligation, and PCR amplification. The library was hybridised overnight with the panel probes. Unbound fragments were then washed away. The quantity and quality of the purified cfDNA were checked using Qubit fluorimeter and Bioanalyzer 2100. Paired-end sequencing with 2 × 150 bp reads was performed using the Illumina sequencing platform. cfDNA testing from plasma was conducted in China (Huidu Shanghai Medical Sciences Ltd.) using the PredicineCARE assay targeting 152 genes. Detailed information can be found in Supplementary Fig. S2.

### Single-cell preparation and sequencing

The collected pT_CD8+CD28-_ and pT_CD8+CD28+_ were separately stored in the sCelLive^TM^ Tissue Preservation Solution (Singleron Biotechnologies, Nanjing, China) and then transported to the Singleron lab on ice as soon as possible. The sample was stained with trypan blue (Sigma, Shanghai, China) and microscopically evaluated for cell viability. Single-cell suspensions at 1 × 10^5^ cells/mL in PBS (HyClone, Shanghai, China) were prepared and loaded onto microfluidic devices and scRNA-seq libraries were constructed according to the GEXSCOPE^®^ protocol using the GEXSCOPE^®^ Single-Cell RNA Library Kit (Singleron Biotechnologies) and Singleron Matrix^®^ Automated single-cell processing system (Singleron Biotechnologies). Individual libraries were diluted to 4 ng/µL and pooled for sequencing. Pools were sequenced on Illumina novaseq6000 with 150-bp paired-end reads.

### Statistical analysis

Clinical data were obtained from the patient electronic medical recording system. The relationships between pT_CD8+CD28-_ levels, mutation prevalence and other clinical characteristics were assessed using the Chi-square test or Fisher exact test, Student *t* test or Mann–Whitney *U* test, and Pearson correlation tests accordingly. The training and validation sets were allocated to each case using computer-generated randomised numbers. All analyses were conducted by SPSS 19.0 version software (IBM Inc., NY, USA). Missing data was excluded from the analysis. The cut-off value of pT_CD8+CD28-_ level regarding PFS was calculated using the software of X-tile 3.6.1 version reported by Camp RL et al. [23] Kaplan–Meier survival analysis and the log-rank test were used to compare PFS between different patient cohorts. The Cox proportional hazard regression model was utilised to estimate the hazard ratio (HR) and 95% confidence interval (CI) of the proportion of pT_CD8+CD28-_ in peripheral blood, while adjusting for confounding factors. All statistical tests were two-sided with a significance level set at 0.05.

## Results

### Basic characteristics of the study cohort

A total of 252 HER2 + MBC patients were enrolled in this study. The clinical characteristics of the cohort are presented in Table 1. In summary, the median follow-up time of the HER2 + MBC cohort was 29.6 months (range: 3.7–95.0 months), and the median age at diagnosis was 52 years (range: 25–82). At the time of treatment, primary stage IV breast cancer was observed in 67 patients (26.6%), while recurrent breast cancer was present in 185 patients (71.9%). All individuals of the cohort received first-line therapy. Out of the 252 patients, 151 (60.0%) received chemotherapy plus trastuzumab, 49 (19.4%) received chemotherapy in combination with trastuzumab and pertuzumab, 23 (9.1%) received chemotherapy plus tyrosine kinases inhibitors (TKIs) and 29 (11.5%) received chemotherapy alone. In those who received TKIs, all had previously undergone adjuvant and/or neoadjuvant anti-HER2 therapy involving trastuzumab and/or pertuzumab.

**Table 1:** Clinical characteristics of the study cohort (*n* = 252).

Clinical characteristics	*n* (%)
Age at diagnosis (range: 25–82, median = 52)	
≤45 years	83 (32.9)
>45 years	169 (67.1)
Primary T stage	
I	60 (23.8)
II	127 (50.4)
III	24 (9.5)
IV	30 (11.9)
Unknown	11 (4.4)
Primary N stage	
0	64 (25.4)
1	59 (23.4)
2	47 (18.7)
3	67 (26.6)
Unknown	15 (5.9)
Primary TNM stage	
I	23 (9.1)
II	73 (29.0)
III	76 (30.2)
IV	67 (26.6)
Unknown	13 (5.1)
Primary tumour grade	
I	6 (2.4)
II	157 (62.3)
III	74 (29.4)
Unknown	15 (5.9)
Site of metastasis	
Liver	93 (36.9)
Lung	97 (38.5)
Brain	12 (4.8)
Bone	93 (36.9)
Lymph node	160 (63.5)
Chest wall	41 (16.3)
Others^a^	33 (13.1)
Number of metastatic sites^b^	
1	90 (35.7)
2–3	132 (52.4)
≥4	30 (11.9)
Disease-free survival (range: 3–283, median = 32)	
≤36 months	104 (56.2)
>36 months	81 (43.8)
Regimens of first-line therapy	
Chemotherapy plus trastuzumab	151 (60.0)
Chemotherapy plus trastuzumab & pertuzumab	49 (19.4)
Chemotherapy plus TKIs^**c**^	23 (9.1)
Chemotherapy alone	29 (11.5)

^a^Other metastasis including pleural, adrenal, cutaneous, intestinal and soft tissue metastasis.

^b^Multiple lesions occurred in the same organ only count once.

^c^Tyrosine kinase inhibitors.

### The prognostic value of baseline pT_CD8+CD28-_ level

**Fig. 2:**
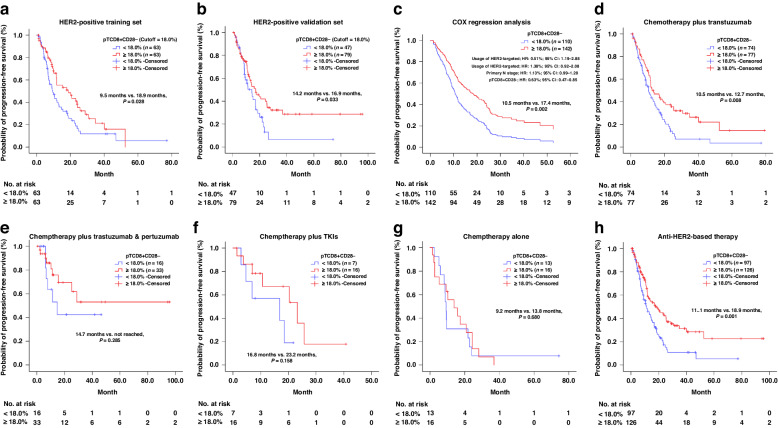
The prognostic role of baseline pTcd8+cd28- in HER2 + MBC patients regarding first-line PFS. Determine the prognostic role of pTcd8+cd28− in training set (**a**) and retest in the validation set (**b**). Cox regression analysis was performed for pTcd8+cd28−, including other factors contributing to the PFS. The HR values for each factor derived from the multivariate analysis are also presented (**c**). The prognostic role of pTcd8+cd28− in the chemotherapy plus trastuzumab subgroup (**d**), in the chemotherapy plus trastuzumab & pertuzumab subgroup (**e**), in the chemotherapy plus TKIs subgroup (**f**) and in chemotherapy-alone subgroup (**g**); The prognostic role of pTcd8+cd28− in patients who received anti-HER2 based therapy (**h**). pTcd8+cd28− peripheral CD8 + CD28- T cell, TKIs tyrosine kinase inhibitors.

The 252 patients with HER2 + MBC were randomly allocated into two groups: the training set (*n* = 126) and the validation set (*n* = 126). Using X-tile software, we evaluated the prognostic potential of each pL subtype in terms of first-line PFS within the training set. Consequently, only the pT_CD8+CD28−_ level demonstrated a significant association with first-line PFS in HER2 + MBC patients (Supplementary Fig. S3). The optimal cut-off value for pT_CD8+CD28-_ was determined to be 18.0% (ranging from 16.5 to 19.0%) with a significant *P* value of 0.028, as shown in Fig. 2a. This identified cut-off value was subsequently validated in the independent validation set (*P* = 0.033) (Fig. 2b). Apart from pT_CD8+CD28-_ level, other baseline characteristics that may contribute to first-line PFS were also assessed through univariate analysis. These characteristics included the primary lymph node stage (*P* = 0.017), the presence of uncommon metastatic sites (*P* = 0.001) and the utilisation of HER2-targeted therapy (*P* = 0.034) (Supplementary Table S2). The final Cox regression model in multivariate analysis was adjusted to include all variables that showed statistical significance in the aforementioned univariate analysis. Consistent with expectations, the Cox regression model confirmed that the baseline pT_CD8+CD28−_ level served as an independent prognostic factor for first-line PFS among HER2 + MBC patients, and high pT_CD8+CD28-_ level (≥18.0%) was associated with a prolonged median PFS (mPFS) compared to low level (10.5 vs. 17.4 months, *P* = 0.002) (Fig. 2c).

### The prognostic value of baseline pT_CD8+CD28-_ level in different therapeutic subgroups

Within the cohort, HER2 + MBC patients were stratified into four subgroups based on their therapeutic regimens. In the subgroup receiving chemotherapy plus trastuzumab (*n* = 151), individuals with of pT_CD8+CD28-_ high exhibited a prolonged mPFS compared to those with lower levels (10.5 vs. 12.7 months, *P* = 0.008), which aligns with our overall cohort findings (Fig. 2d). However, the significant difference in mPFS between pT_CD8+CD28-_ high and pT_CD8+CD28-_ low was not found in the subgroups receiving chemotherapy in combination with trastuzumab and pertuzumab (not reached vs. 14.7 months, *P* = 0.285), chemotherapy plus tyrosine kinase inhibitor (TKI) subgroup (16.8 vs. 23.2 months, *P* = 0.158) and chemotherapy-alone subgroup (9.2 vs. 13.8 months, *P* = 0.680) (Fig. 2e–g). Nevertheless, for HER2-targeting-based therapy (*n* = 223), the pT_CD8+CD28-_ High at baseline was associated with prolonged mPFS (11.1 vs. 18.9 months, *P* = 0.001) (Fig. 2h).

### The prognostic value of dynamic pT_CD8+CD28−_ levels

**Fig. 3:**
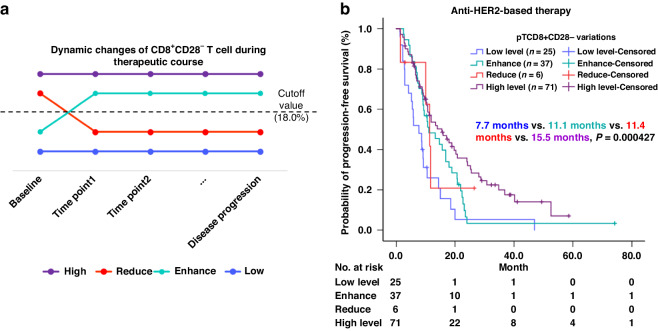
The prognostic value of dynamic change of pTcd8+cd28- regarding first-line PFS. The diagram for grouping dynamic change of pTcd8+cd28− (**a**). The prognostic role of dynamic change of pTcd8+cd28− was assessed using K–M analysis (**b**).

Considering the aforementioned results, we sought to determine the relationship between the dynamic alteration of pT_CD8+CD28-_ and first-line PFS. Based on variations in pT_CD8+CD28-_, patients who underwent anti-HER2-based therapy were categorised into four groups: high-level, low-level, reducing and enhancing groups respectively. The high-level group consistently maintained a high level of pT_CD8+CD28-_ throughout the entire therapeutic course, while the low-level group consistently maintained a low level. The reducing group initially exhibited a high level but subsequently showed a low level at each follow-up visit, whereas the enhancing group demonstrated the opposite pattern (Fig. 3a). Consequently, we included a total of 139 patients who met these criteria in Kaplan–Meier analysis regarding first-line median PFS. Among them, the high-level group (*n* = 71) had the longest mPFS of 15.5 months, while the low-level group (*n* = 25) had the shortest first-line mPFS of 7.7 months. Meanwhile, both the reducing group (*n* = 6, mPFS = 11.4 months) and the enhancing group (*n* = 37, mPFS = 11.1 months) displayed similar mPFS durations. In summary, we demonstrated that the dynamic changes in pT_CD8+CD28-_ levels were also associated with first-line PFS in patients with HER2 + MBC who received anti-HER2-based therapy (*P* = 0.000427) (Fig. 3b). However, it is important to note that this prognostic value was observed within a limited sample size (*n* = 139); therefore, further validation in an independent cohort is warranted for future studies.

### Association of pT_CD8+CD28-_ level with other clinical characteristics

In addition to assessing the prognostic value of pT_CD8+CD28-_, we also investigated its correlation with other clinical characteristics in the cohort. The baseline pT_CD8+CD28-_ level exhibited a significant negative correlation with bone metastasis (coefficient = −0.189, *P* = 0.003), lymph node metastasis (coefficient = −0.152, *P* = 0.016), and the number of metastatic sites (coefficient = −0.200, *P* = 0.001) (Table 2). Furthermore, there was a negative correlation between changes in pT_CD8+CD28-_ and bone metastasis (coefficient = −0.218, *P* = 0.006) (Supplementary Table S3). Notably, both bone and lymph node are all critical immune-related organs. Compared to health controls, HER2 + MBC patients exhibited a unique positive correlation between pT_CD8+CD28-_ and total T cells (coefficient = 0.134, *P* = 0.033) as well as natural killer T cells (coefficient = 0.143, *P* = 0.024) (Table 3). These findings, however, require further validation and should be cautiously interpreted due to the low coefficients.

**Table 2:** Association of pT_CD8+CD28−_ with the clinical characteristics.

Clinical characteristics	Correlation coefficient	*P* value
Age of diagnosis	0.030	0.633
≤45 years (*n* = 83)		
>45 years (*n* = 169)		
Age of sample collection	0.022	0.723
Primary T stage	0.024	0.697
I (*n* = 59)		
II (*n* = 122)		
III (*n* = 22)		
IV (*n* = 28)		
Primary N stage	0.026	0.668
0 (*n* = 64)		
1 (*n* = 59)		
2 (*n* = 47)		
3 (*n* = 67)		
Primary tumoral grade	−0.026	0.703
I (*n* = 5)		
II (*n* = 144)		
III (*n* = 68)		
Liver	−0.123	0.052
No (*n* = 159)		
Yes (*n* = 93)		
Lung	−0.011	0.864
No (*n* = 155)		
Yes (*n* = 97)		
Brain	−0.104	0.100
No (*n* = 240)		
Yes (*n* = 12)		
Bone	−0.189	0.003
No (*n* = 159)		
Yes (*n* = 93)		
Lymph	−0.152	0.016
No (*n* = 92)		
Yes (*n* = 160)		
Chest	0.063	0.320
No (*n* = 211)		
Yes (*n* = 41)		
Uncommon metastatic lesion	−0.085	0.177
No (*n* = 219)		
Yes (*n* = 33)		
Visceral metastasis	−0.081	0.198
No (*n* = 89)		
Yes (*n* = 163)		
Number of metastatic sites	−0.200	0.001
1 (*n* = 90)		
2–3 (*n* = 132)		
≥4 (*n* = 30)		
DFS	−0.103	0.156
≤36 months (*n* = 104)		
>36 months (*n* = 81)		
Application of anti-HER2 therapy	0.009	0.892
No (*n* = 29)		
Yes (*n* = 223)	0.030

**Table 3:** Correlation of pT_CD8+CD28−_ level with other peripheral lymphocyte subtypes.

Peripheral lymphocyte subtypes	HER2 + MBCs (*n* = 252)		Health control (*n* = 79)	
	Correlation coefficient	*P* value	Correlation coefficient	*P* value
CD3+ (total T cell)	0.134	0.033	0.079	0.486
CD3 + CD4+ (T helper cell)	−0.274	<0.001	−0.251	0.026
CD3 + CD8+ (cytotoxic T cell)	0.422	<0.001	0.441	<0.001
CD4 + /CD8+ ratio	−0.474	<0.001	−0.443	<0.001
CD3-CD16 + CD56+ (natural killer cell)	0.099	0.116	0.134	0.238
CD3 + CD16 + CD56+ (natural killer T cell)	0.143	0.024	0.014	0.904
CD19+ (B cell)	−0.199	0.002	−0.196	0.028
CD4 + CD25 + T cell	−0.260	<0.001	−0.154	0.175
CD8 + CD28+ (naive antigen-specific T cell)	−0.297	<0.001	−0.287	0.010

### The cytotoxic potential of pT_CD8+CD28-_ in HER2-positive MBC

**Fig. 4:**
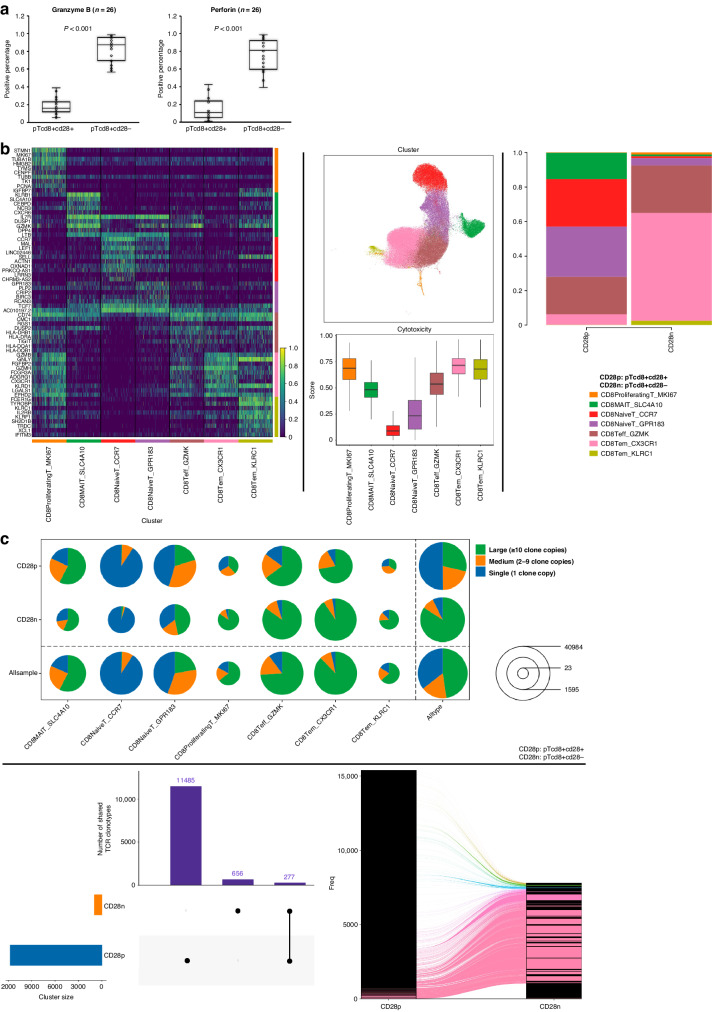
The assessment of the cytotoxic capacity of pTcd8+cd28-. Detection of expression of Perforin and Granzyme B in pTcd8+cd28- and pTcd8+cd28+ (**a**). Comparison of cell cluster composition between pTcd8+cd28- and pTcd8+cd28+ using single-cell RNA sequencing (**b**). Cell cluster annotation based on well-known cell markers (**b**, left). Cytotoxic score evaluation of annotated T-cell clusters (**b**, middle). Cell cluster distributions of pTcd8+cd28- and pTcd8+cd28+ (**b**, right); TCR repertoire comparison between pTcd8+cd28- and pTcd8+cd28+ (**c**). TCR clonality distribution in pTcd8+cd28- and pTcd8+cd28+ (**c**, upper). The shared clones between pTcd8+cd28- and pTcd8+cd28+ and clone expansion in pTcd8+cd28- (**c**, lower).

To identify the in vivo cytotoxic potential of pT_CD8+CD28−_, we assessed the secretion of cytotoxic effectors, T-cell receptor (TCR) clonality, and transcriptome of pT_CD8+CD28-_ derived from the enrolled patients. Initially, significantly higher levels of perforin (84.29 ± 3.3 vs. 19.14 ± 2.0%, *P* <0.001) and granzyme B (76.10 ± 4.2 vs. 16.65 ± 2.4%, *P* <0.001) were observed in pT_CD8+CD28-_ in comparison to its precursor cell (pT_CD8+CD28+_) (Fig. 4a). Using single-cell RNA sequencing, seven T-cell subtypes were identified across pT_CD8+CD28+_ and pT_CD8+CD28-_, based on well-known expression patterns of cell markers (Fig. 4b, left). As compared to pT_CD8+CD28+_, the predominant cell subtypes of pT_CD8+CD28-_ composed of CD8+ effector T cells and CD8+ effector memory T cells (92.48% vs. 27.92%) (Fig. 4b, right). These two subtypes exhibited higher cytotoxic scores among others (Fig. 4b, middle), indicating the cytotoxicity of pT_CD8+CD28-_. TCR repertoire analysis further revealed a lower diversity-higher clonality of pT_CD8+CD28-_ as compared to pT_CD8+CD28+_, particularly for those effector T-cell subtypes of pT_CD8+CD28-_ (Fig. 4c, upper). Meanwhile, in pT_CD8+CD28-_, we observed the expansion of most shared TCR clones between pT_CD8+CD28+_ and pT_CD8+CD28-_ as shown in the Sankey diagram (Fig. 4c, lower). Collectively, the aforementioned data demonstrate that pT_CD8+CD28-_ functions as an antigen-experienced effector T cell in HER2 + MBC.

### Association of pT_CD8+CD28-_ with the tumour immunity

**Fig. 5:**
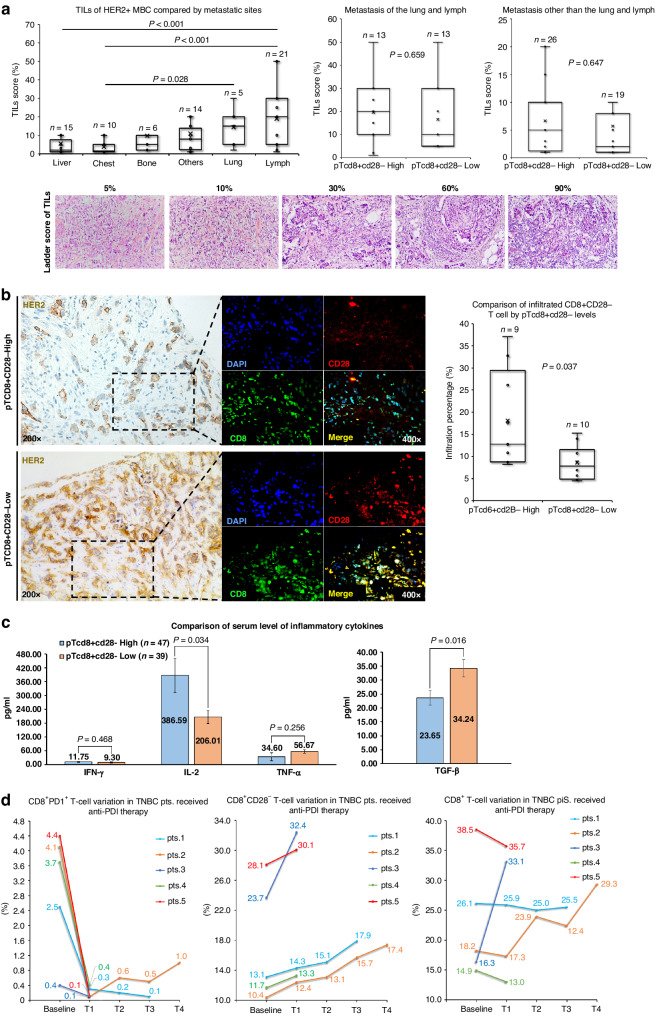
The association of pTcd8+cd28- level with tumour immunity. Comparison of tumour-infiltrating lymphocyte level based on metastatic sites (**a**, left) and pTcd8+cd28- levels across different metastatic sites (**a**, right); Comparison of infiltrated CD8 + CD28- T cells from patients with pTcd8+cd28- high and pTcd8+cd28- low (**b**). Serum levels of IFN-γ, IL-2, TNF-α, and TGF-β in patients with pTcd8+cd28- high and pTcd8+cd28- low (**c**). The changes in peripheral CD3 + CD8 + T cells (left), CD8 + PD1 + T cells (middle) and CD8 + CD28- T cells (right) following combination therapy with anti-PD1 and MASCT (**d**).

To ascertain the relationship between pT_CD8+CD28-_ levels and patients’ tumour immunity, we initially compared the TILs infiltration in individuals with high and low levels of pT_CD8+CD28-_. Considering the variability in TILs infiltration among different metastatic sites in HER2 + MBC (Fig. 5a, left), we separately analysed the TILs scores as depicted in right panel of Fig. 5a. Despite observing higher TILs scores among patients with pT_CD8+CD28-_ high in the lymph and/or lung metastasis group (19.6 ± 3.9 vs. 16.5 ± 4.0%) and other metastasis sites group (6.7 ± 4.8 vs. 5.7 ± 1.2%), none of these differences reached statistical significance (Fig. 5a, right). Confocal immunofluorescence analysis revealed a positive quantitative correlation between pT_CD8+CD28-_ and infiltrated CD8 + CD28- T cells in the paired tumour lesions (*P* = 0.037, Fig. 5b, right). We then compared the serum level of various inflammatory cytokines between high and low level of pT_CD8+CD28-_. Patients with pT_CD8+CD28-_ high exhibited elevated IL-2 levels (386.6 ± 73.7 vs. 206.0 ± 28.5 pg/ml, *P* = 0.034) and decreased TGF-β levels (23.7 ± 2.7 vs. 34.2 ± 3.1 ng/ml, *P* = 0.016) comparing to pT_CD8+CD28-_ low. However, no statistical differences were observed for IFN-γ or TNF-α (Fig. 5c). In addition, cfDNA-based sequencing revealed a higher prevalence of *CD274* (PD-L1) deletion in pT_CD8+CD28-_ high as compared to pT_CD8+CD28-_ low (*P* = 0.041, Supplementary Fig. S4).

To further substantiate the association between pT_CD8+CD28-_ and tumour immunity, we investigated the variations of pT_CD8+CD28-_ mediated by immunotherapy. Regrettably, immunotherapy is not currently employed as a standard regimen for HER2 + MBC patients. Alternatively, we evaluated our hypothesis in the context of metastatic triple-negative breast cancer (mTNBC) patients who are eligible for immunotherapy. We enrolled five mTNBCs patients received combination therapy of anti-PD1 and multiple antigen-specific cell therapy (MASCT) at different therapeutic lines. Specifically, patients with PD-L1-positive tumours underwent a dose-escalation study using intravenous camrelizumab (3 mg/kg, every 2 weeks), along with intravenous infusion of T cells and dendritic cells (DCs) every 27–36 days. Meanwhile, the baseline and subsequent follow-up assessments until disease progression revealed a decrease in peripheral levels of CD8 + PD1 + T cells (Fig. 5d, left) and an increase in pT_CD8+CD28-_ levels (Fig. 5d, middle) across all five patients. In contrast, the fluctuation in peripheral CD3 + CD8 + T-cell levels did not demonstrate a consistent pattern, thereby ruling out the possibility of false-positive amplification of pT_CD8+CD28-_ due to T-cell infusion (Fig. 5d, right).

## Discussion

Despite the dramatic clinical improvements during the past two decades, resistance to HER2-targeted therapy remains virtually inevitable [24]. Therefore, there is an urgent need for a robust predictor of therapeutic efficacy. Numerous studies have reported biomarkers associated with the effectiveness of anti-HER2 therapy [25, 26]. Recently, immune-related factors have garnered much attention among a range of biomarkers. In 2023, Hills RK and his colleagues reported the prognostic value (but not the predictive value) of TILs in early breast cancer patients receiving trastuzumab [27]. In metastatic setting, TILs also exhibit a prognostic value for OS, as evidenced by a retrospective analysis of the CLEOPATRA trial [28]. These findings suggest a connection between tumour immunity and the overall outcome of the patients. The detection of peripheral lymphocytes offers advantages such as real-time monitoring, minimal invasiveness, and homogeneity compared to needle biopsy-based detection. Here, we demonstrated the prognostic value of pT_CD8+CD28-_ in MBC patients receiving anti-HER2-based therapy. At baseline, patients with pT_CD8+CD28-_ high had significantly longer mPFS than those with pT_CD8+CD28-_ low (Fig. 2a–c). In subgroup analysis, the same result was reproduced in patients who received trastuzumab plus chemotherapy (Fig. 2d). Although similar trends were noted in other subgroups, none of these reached statistical significance (Fig. 2e–g). This may be partly due to the limited sample size and the insufficient follow-up time period. Moreover, the dynamic monitoring of pT_CD8+CD28-_ also showed a prognostic value comparable to the baseline pT_CD8+CD28-_ level (Fig. 3). It is noteworthy that even among patients with pT_CD8+CD28-_ low at baseline, those who exhibited an increased pT_CD8+CD28-_ level upon HER2-targeting therapy also gained more benefit than those maintain pT_CD8+CD28-_ low (7.7 vs. 11.1 months, *P* = 0.025) (Fig. 3). Evidence have demonstrated the anti-tumour effect of trastuzumab induced antibody-dependent cellular cytotoxicity (ADCC) [29]. Therefore, we hypothesize that pT_CD8+CD28-_ level may reflect tumour immunity of the patients, thus aiding in the identification of individuals who are more likely to benefit from HER2-targeted therapy. Numerous studies have highlighted the suppressive role of CD8 + CD28- T cells in regulating tumour immunity, suggesting that the presence of cell surface markers CD8+ and CD28- signifies a senescent or exhausted phenotype of T cell [30]. According to our data, however, high level of pT_CD8+CD28-_ appears to indicate a positive tumour immunity in HER2 + MBC. First, we found an intensive expression of perforin and granzyme B in pT_CD8+CD28-_, suggesting its cytotoxic function (Fig. 4a). In addition, TCR clonality and transcriptome analysis further revealed that pT_CD8+CD28-_ served as an antigen-experienced effector T cell in HER2 + MBC (Fig. 4b, c). In peripheral blood, pT_CD8+CD28-_ level showed a positive correlation with total T cells and nature killer T-cell level (Table 3). In metastatic lesions, we noticed a trend indicating that pT_CD8+CD28-_ level was positively associated with TILs infiltration, particularly CD8 + CD28− T-cell infiltration (Fig. 5a, b). The maintenance of terminally differentiated effector T cells is well-known to be dependent on IL-2 and can be disrupted by transforming growth factor-β (TGF-β) [31, 32]. Our findings support this notion, as we found a significant association between a higher serum level of IL-2 (*P* = 0.034) and a lower level of TGF-β (*P* = 0.016) with pT_CD8+CD28-_ high (Fig. 5b). These evidence were consistent with what we found at cellular level. Furthermore, we also observed the anti-PD1 therapy induced upregulation of pT_CD8+CD28-_ level, although this result was achieved in TNBC patients (Fig. 5). This clinical phenomenon further strengthens our hypothesis regarding the close relationship between pT_CD8+CD28-_ and tumour immunity. Still, our hypothesis needs more detailed investigation and concrete evidence. Collectively, we demonstrated that a high level of pT_CD8+CD28-_ is associated with enhanced tumour immunity in HER2 + MBC. This observation provides partial insight into why patients with an intensive or increasing level of pT_CD8+CD28-_ upon anti-HER2-based therapy could gain more benefit.

There are several limitations of present study. Firstly, this retrospective study has a limited number of patients in most therapeutic subgroups. Perspective studies designed to verify the predictive value of pT_CD8+CD28-_ for each therapeutic subgroup are necessary. Secondly, the observed increase in pT_CD8+CD28-_ level following anti-PD1 therapy in mTNBC patients should also be validated in HER2 + MBC patients. Finally, the comprehensive mechanisms underlying the prognostic role of pT_CD8+CD28-_ and its effect on tumour progression remain unclear, necessitating further exploration.

## Conclusions

Our study has identified the novel prognostic value of pT_CD8+CD28-_ for anti-HER2-based therapy in MBC patients. Specifically, patients with pT_CD8+CD28-_ high at baseline exhibited prolonged PFS. Patients with consistent or enhanced pT_CD8+CD28-_ levels upon the treatment also indicate a better PFS. In addition, we observed a positive correlation between pT_CD8+CD28-_ levels and tumour immunity, which can be enhanced by immunotherapy.

### Supplementary information

Supplemental data

## Data Availability

Data generated during this study are available from the corresponding author upon reasonable request.

## Materials availability

Materials generated during this study are available from the corresponding author upon reasonable request.
