# Supplementary material for: Persistence of peripheral CD8 + CD28− T cells indicates a favourable outcome and tumour immunity in first-line HER2-positive metastatic breast cancer
Source: Br J Cancer. 2024 Mar 22;130(10):1599–608. doi: 10.1038/s41416-024-02610-0 (PMC11091143; doi:10.1038/s41416-024-02610-0)
Supplement: Supplementary file 1 — Supplemental data [file 41416_2024_2610_MOESM1_ESM.pdf]

Figure S1

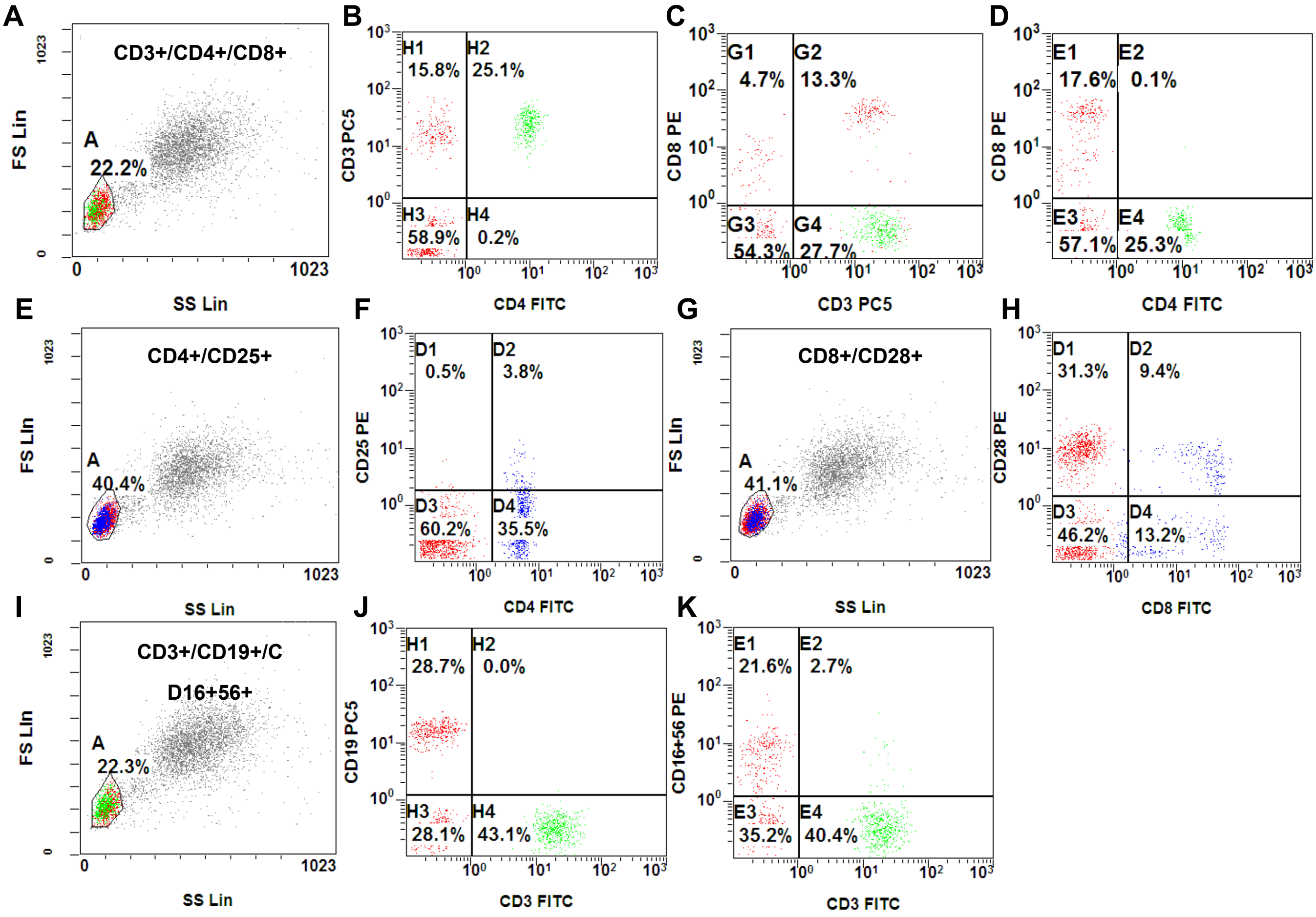

Figure S1. The gating strategy employed for the cytometric analysis of each peripheral lymphocyte subtypes. Using typical forward and side scatter characteristics, a gate was set on lymphocytes expressed CD3 and/or CD4 and/or CD8 (A); Within the CD3+/CD4+/CD8+ gate, Helper T-cells were determined by gating on CD3+ & CD4+ (B); Cytotoxic T-cell were determined by gating on CD3+ & CD8+ phase (C); The CD4/CD8 ratio was determined by gating on CD4+ & CD8+ (E4 phase/E1 phase)(D); A new gate was set on lymphocytes expressed CD4 and/or CD25 (E); Within the CD4+/CD25+ gate, CD4+CD25+ T cell were determined by gating on CD4+ & CD25+ phase (F); Another gate was set on lymphocytes expressed CD8 and/or CD28 (G); Within the CD8+/CD28+ gate, Activated naive cytotoxic T-cells were determined by gating on CD8+ & CD28+ phase, and pTcd8+cd28- were determined by gating on CD8+ & CD28- phase (H); Lymphocytes expressed CD3 and/or CD19 and/or both CD16 and CD56 were isolated using preset gate as showed in fig. S2A; Within the CD3+/CD19+/CD16+56+ gate (I), B cells were determined by gating on CD3- & CD19+ phase (J); Natural killer cells were determined by gating on CD3- & CD16+56+ phase, and Natural killer T-cell were determined by gating on CD3+ & CD16+56+ phase (K).

Gene List & Performance Specifications

Figure S2

PredicineCARE™ interrogates 152 key genes

ABRAXAS1	AKT1	AKT2	AKT3	ALK	APC	AR	ARAF	ARID1A	ATM
ATRX	BAP1	BARD1	BCL2	BRAF	BRCA1	BRCA2	BRIP1	BTK	CCND1
CCND2	CCND3	CCNE1	CCNE2	CD274 (PD-L1)	CD74	CDH1	CDK12	CDK2	CDK4
CDK6	CDKN2A	CHEK1	CHEK2	CTNNB1	CXCR4	CYP2C19	CYP2D6	CYP3A4	DAXX
DDR2	DPYD	E2F1	EGFR	EPCAM	ERBB2 (HER2)	ERBB3	ERCC1	ESR1	EZH2
FANCA	FANCC	FANCF	FANCG	FANCL	FAT1	FBXW7	FEN1	FGFR1	FGFR2
FGFR3	FGFR4	FLT3	FOXA1	FOXL2	FZR1	GEN1	GNA11	GNAQ	GNAS
GSTP1	HNF1A	HOXB13	HRAS	IDH1	IDH2	JAK2	JAK3	KDM6A	KIT
KMT2C	KMT2D (MLL2)	KRAS	MAP2K1 (MEK1)	MAP2K2 (MEK2)	MAPK1	MAPK3	MDM2	MET	MLH1
MPL	MRE11	MSH2	MSH6	MTHFR	MTOR	MYC	MYCN	MYD88	NBN
NF1	NFE2L2	NOTCH1	NPM1	NRAS	NTRK1	NTRK2	NTRK3	PALB2	PDCD1LG2 (PD-L2)
PDGFRA	PIK3CA	PIK3CB	PIK3R1	PLCG2	PMS2	POLD1	POLE	PPP2R1A	PRKACA
PRKD1	PTEN	PTPN11	RAD50	RAD51	RAD51B	RAD51C	RAD51D	RAD52	RAF1
RB1	RET	RHEB	RHOA	RIT1	RNF43	ROS1	SDHB	SMAD4	SMO
SPOP	STAG2	STK11	TERT promoter	TMPRSS2	TP53	TSC1	TSC2	UGT1A1	VHL
XPC	XRCC1								

SNVs + Indels CNVs Fusions Fusions + CNVs

PERFORMANCE SPECIFICATIONS

	Reportable Range	Allele Frequency/Copy Number	Sensitivity	Positive Predictive Value (PPV)
Single Nucleotide Variations	≥0.05%	≥0.5% AF	100%	100%
		0.25% - 0.5% AF	98.6%	99.2%
		<0.25% AF	78.3%	97.9%
Indels	≥0.05%	≥0.5% AF	100%	100%
		0.25% - 0.5% AF	98.6%	100%
		<0.25% AF	80%	100%
Re-arrangement	≥0.05%	≥0.5% AF	100%	100%
		0.375 - 0.5% AF	96.7%	100%
		0.25% - 0.375% AF	90%	100%
		<0.25% AF	33.3%	100%
Copy Number Gain	≥2.18	≥2.375 copies	100%	100%
		2.23 - 2.375 copies	100%	100%
		<2.23 copies	45%	81.8%
Copy Number Loss	≤1.85	≤1.75 copies	100%	100%
		1.75 - 1.80 copies	93.6%	91.7%
		1.80 - 1.85 copies	66%	88.6%
Sequencing and Bioinformatics	Illumina NGS			
Turnaround Time	10 days			
Target Sequence Coverage	20,000x for biofluid, 2,000x for tissue			
Specimen Type and Requirement		CLIA	RUO	
	Liquid biopsy (blood)	8 mL plasma<br>2 tubes of whole blood	2-4 mL plasma<br>1 tube of whole blood	
	Liquid biopsy (urine)	20-40 mL urine	40 mL urine	
	Tissue biopsy	Not applicable	2-10 FFPE slides	

Figure S2. Detected Gene list and mutational type of 152-gene PredicineCARE microarray

Figure S3

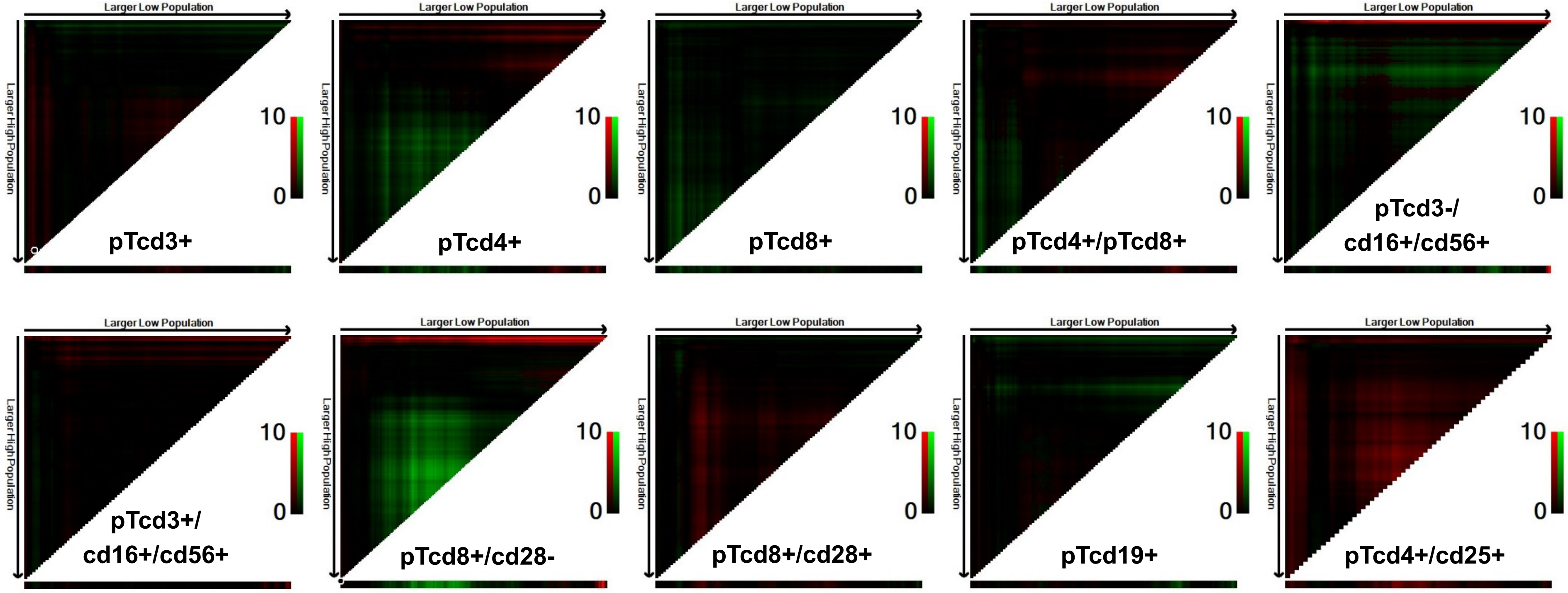

Figure S3. The prognostic value of 10 peripheral lymphocyte subtypes regarding PFS in training set were evaluated using X-tile software. The X-axis represents a range of potential cut-points, arranged from low to high (left to right). Conversely, the Y-axis represents a range of cut-points, arranged from high to low (top to bottom). Red pixel indicates an inverse correlation with PFS, whereas green pixel represents positive correlation. The most favorable cut-off value can be identified at the region with the highest luminosity (green or red).

Figure S4

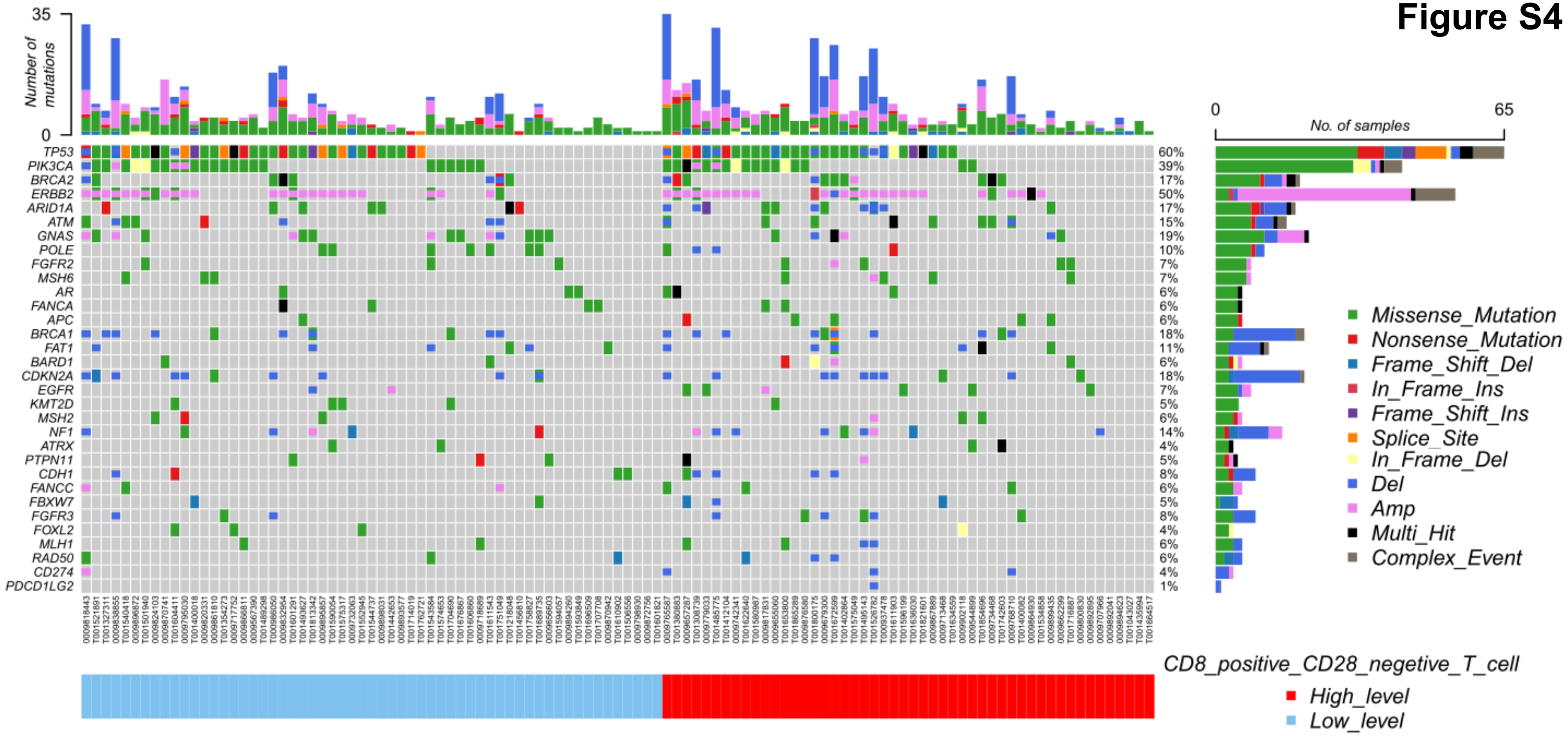

Figure S5. Comparison of cell free DNA based mutation landscape between patients with pTcd8+cd28- high and pTcd8+cd28- low.

Table S1. Selection criteria of the study

Selection criteria	
**Inclusion**	**Exclusion**
Age > 18 years	Any microbial infection especially for cytomegalovirus
Pathologically confirmed recurrent and metastatic breast cancer	Autoimmune disease
IHC or FISH confirmed HER2 over-expression/amplification	Concurrent hematological disorders and other malignancies
No anti-tumor therapy after relapse or prior to enrollment	Loss of follow-up within 3 months after enrollment
Eligible to receive standard HER2-targeted therapy and/or chemotherapy	Patients who demanded withdrawal from the study
Expected survival time of 3 months or beyond	
Provision of written and informed consent	

Table S2. Multivariate analysis regarding first-line PFS

**Baseline Characteristics**	**HR (95%CI)**	***P* value**
Age of diagnosis (Cutoff=45years)	0.89 (0.67-1.20)	0.446
Primary T stage	1.11 (0.94-1.30)	0.218
Primary N stage	2.04 (1.13-3.68)	0.017
Primary tumor grade	1.05 (0.76-1.44)	0.781
Liver metastasis	0.95 (0.72-1.27)	0.748
Lung metastasis	1.22 (0.92-1.63)	0.163
Brain metastasis	1.11 (0.98-1.26)	0.091
Bone metastasis	1.11 (0.84-1.48)	0.466
Lymph metastasis	1.13 (0.84-1.51)	0.416
Chest metastasis	1.13 (0.78-1.63)	0.512
Uncommon metastasis	1.95 (1.34-2.84)	0.001
Visceral metastasis	1.11 (0.61-3.82)	0.485
Number of metastatic sites	0.71 (0.47-1.06)	0.094
DFS (Cutoff=36.0 months)	1.02 (0.74-1.42)	0.897
First-line regimen	1.04 (0.58-1.19)	0.583
Application of anti-HER2 therapy	0.49 (0.27-0.88)	0.034
CD8+CD28- (Cutoff=18.0%)	0.66 (0.49-0.87)	0.003

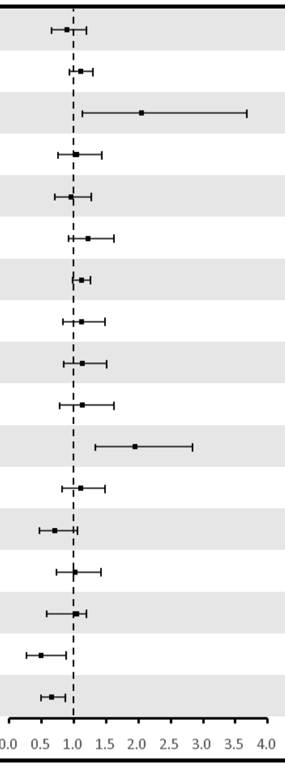

The HR value of each clinical characteristic is derived from univariate analysis.

Table S3. Clinical characteristics that are significantly associated with $pT_{CD8+CD28-}$ variation

Clinical characteristics	$pT_{CD8+CD28-}$ variations				Correlation coefficient	*P* value
	maintain low (%)	enhance (%)	reduce (%)	maintain high (%)		
**Bone metastasis**					-0.218	0.006
No (n=90)	16 (17.8)	16 (17.8)	2 (2.2)	56 (62.2)		
Yes (n=49)	9 (18.4)	21(42.9)	4(8.2)	15 (30.6)		
**Brain metastasis**					-0.207	0.010
No (n=132)	21 (15.9)	16 (26.5)	2 (4.5)	56 (53.0)		
Yes (n=7)	4 (57.1)	2(28.6)	0(0.0)	1 (14.3)		
